# Impact of the Purification Process on the Spray-Drying Performances of the Three Families of Lipopeptide Biosurfactant Produced by *Bacillus subtilis*

**DOI:** 10.3389/fbioe.2021.815337

**Published:** 2021-12-22

**Authors:** Antoine Vassaux, Marie Rannou, Soline Peers, Théo Daboudet, Philippe Jacques, François Coutte

**Affiliations:** ^1^ Université de Lille, UMRt BioEcoAgro 1158-INRAE, Équipe Métabolites Secondaires d’Origine Microbienne, Institut Charles Viollette, Lille, France; ^2^ LIPOFABRIK, Villeneuve d’Ascq, France; ^3^ TERRA Teaching and Research Centre, Université de Liège, UMRt BioEcoAgro 1158-INRAE, Équipe Métabolites Secondaires D’origine Microbienne, MiPI, Gembloux Agro-Bio Tech, Gembloux, Belgium

**Keywords:** spray-drying, *Bacillus subtilis*, antimicrobial lipopeptides, biosurfactants, surfactin, fengycin, mycosubtilin

## Abstract

Lipopeptides produced by *Bacillus subtilis* display many activities (surfactant, antimicrobial, and antitumoral), which make them interesting compounds with a wide range of applications. During the past years, several processes have been developed to enable their production and purification with suitable yield and purity. The already implemented processes mainly end with a critical drying step, which is currently achieved by freeze-drying. In this study, the possibility to replace this freeze-drying step with a spray-drying one, more suited to industrial applications, was analyzed. After evaluating their thermal resistance, we have developed a spray-drying methodology applicable for the three lipopeptides families produced by *B. subtilis*, i.e., surfactin, mycosubtilin (iturin family), and plipastatin (fengycin family). For each lipopeptide, the spray-drying procedure was applied at three steps of the purification process by ultrafiltration (supernatant, diafiltered solution, and pre-purified fraction). The analysis of the activities of each spray-dried lipopeptide showed that this drying method is not decreasing its antimicrobial and biosurfactant properties. The methodology developed in this study enabled for the first time the spray-drying of surfactin, without adjuvants’ addition and regardless of the purification step considered. In the case of fengycin and mycosubtilin, only diafiltered solution and purified fraction could be successfully spray-dried without the addition of adjuvant. Maltodextrin addition was also investigated as the solution for the direct drying of supernatant. As expected, the performances of the spray-drying step and the purity of the powder obtained are highly related to the purification step at which the product was dried. Interestingly, the impact of mycosubtilin concentration on spray-drying yield was also evidenced.

## Introduction

Secondary metabolites, including those synthesized by multi-enzymatic modular complexes such as polyketide synthase (PKS), non-ribosomal peptide synthetase (NRPS), and hybrid PKS/NRPS structure, represent an almost inexhaustible source of interesting bioactive compounds (Demain, 2014). Non-ribosomal peptides (NRPs) notably show a large structural diversity mainly due to the diversity of monomers, which can compose them (proteogenic and nonproteogenic amino acids, carbohydrates, lipids, etc.). NRPs showing a fatty acid chain linked to a peptide moiety are classified as lipopeptides (LPPs) (Caboche et al., 2008).

**FIGURE 1:**
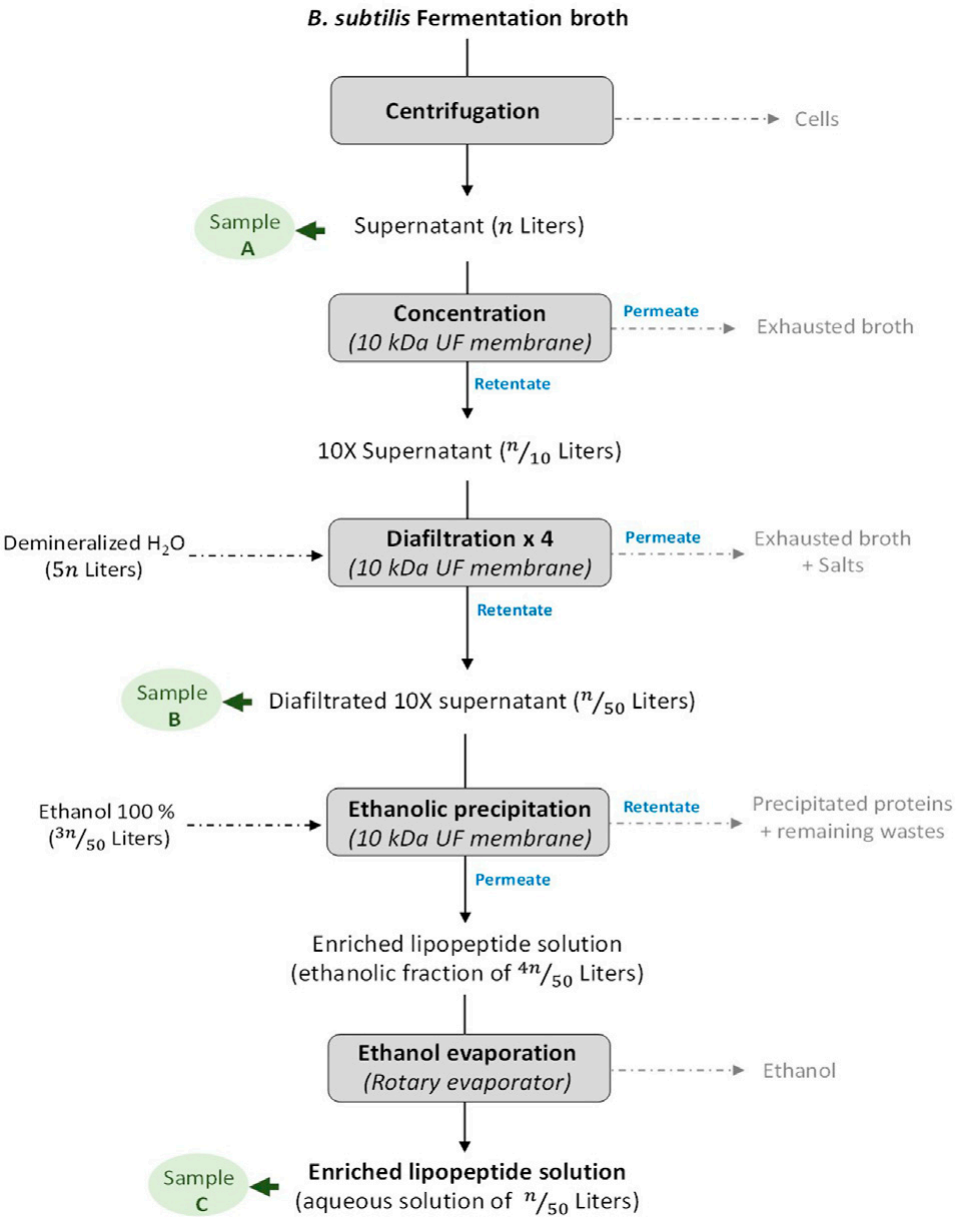
Description of the workflow for the production and purification of lipopeptides from the fermentation of *Bacillus subtilis* to the enriched lipopeptide solution in an aqueous solution. The different steps where a sample has been taken for spray-drying experiments are highlighted as Sample **(A–C)**.

*Bacillus subtilis* is naturally producing and secreting three different families of LPPs: surfactins, iturins, and fengycins. Surfactins (surfactins, pumilacidins, and lichenysins) are cyclic heptapeptides linked to a β-hydroxy fatty acid of C_12_ to C_17_, known for their biosurfactant properties but also for their antiviral and antitumoral activities. Iturins (iturins A, C, D, and E; mycosubtilins; bacillomycins D, F, and L; and mojavensin) consist in a heptapeptide linked to β-amino fatty acid of C_14_ to C_19_, whereas fengycins (fengycins A and B, plipastatins A and B, and agrastatins) show a C_12_ to C_19_ β-hydroxy fatty acid (saturated or not) linked to a decapeptide (Jacques, 2011; Coutte et al., 2017). In addition to their biosurfactant properties, fengycins and iturins are mainly studied for their antifungal properties (Jacques, 2011). LPPs as iturins and fengycins display antifungal properties and are thus used as biocontrol agents to fight against phytopathogens. Several studies have already demonstrated their efficiency on plant fungal pathogens such as *Bremia lactucae* (Deravel et al., 2014), *Zymoseptoria tritici* (Mejri et al., 2018), and *Venturia inaequalis* (Desmyttere et al., 2019) and on foodborne pathogens such as *Paecilomyces variotii*, *Byssochlamys fulva*, and *Candida krusei* (Kourmentza et al., 2021). On the other hand, the elicitor potential of surfactin and its ability to act in synergy with the two other families of LPPs also make it a good candidate for an efficient biocontrol agent (Ongena and Jacques, 2008). The application of these LPPs as biocontrol agents is highly dependent on our capacity to produce and purify them with high yields, suitable purities, and low costs. To achieve this goal, several research teams have already developed innovative processes, such as the disc bioreactor, the membrane bioreactor, or the trickle-bed biofilm reactor, enabling notably to limit the foam apparition due to the surfactant properties of LPPs (Coutte et al., 2017). Conversely, some studies focused on the foaming properties of LPPs to set up overflowing culture processes (Chen et al., 2006; Gong et al., 2009; Guez et al., 2021). In terms of purification, a downstream process encompassing several steps of ultrafiltration/diafiltration/evaporation has already been established and proved its worth in several studies (Coutte et al., 2010a; Kourmentza et al., 2021) (Figure 1). The final formulation of LPPs in powder form is an optional step that enables to concentrate the product to facilitate storage, while reducing the risk of product degradation, thus increasing its shelf-life. In surfactin, iturin, and fengycin cases, the transition from a liquid to a solid state is conventionally operated through a freeze-drying process (Coutte et al., 2010a; Kourmentza et al., 2021).

To dry compounds of interest, freeze-drying and spray-drying are the most used techniques in industrial processing. Due to its lower operating cost and its higher volume capacity, the spray-drying technique is often preferred by food and cosmetics industries (Vardanega et al., 2019). In a recent techno-economic study, authors have highlighted, in one of their scenarios, the requirement of a spray-drying step to set up a surfactin and lichenysin industrial-scale production derived from *Bacillus* fermentation (Czinkóczky and Németh, 2020). Therefore, to fit with industrial requirements, developing a methodology enabling LPPs to dry through spray-drying is of prime interest. The spray-drying technique has already been successfully used to formulate *B. subtilis* strains and its associated culture broth and led to a biocontrol product displaying long shelf-life, high viability, and capability to prevent peach brown rot and rice blast diseases (Yánez-Mendizábal et al., 2012; Meng et al., 2015). The antimicrobial LPPs produced by *Bacillus amyloliquefaciens* were encapsulated by spray-drying, with a limited loss of activity, thanks to the addition of maltodextrin and porous starch as composite wall materials (Wang et al., 2014). Regarding *B. subtilis* LPPs, a surfactin-like biosurfactant, isolated from B30 strain was reported to be successfully spray-dried after a purification step by acid precipitation (Al-Wahaibi et al., 2014). Iturin A produced by *B. subtilis* was also spray-dried to be incorporated into microcapsules, with sodium alginate and poly(γ-glutamic acid) as wall materials, to improve the compound stability and facilitate its storage (Yu et al., 2017). The addition of a high amount of maltodextrin or kaolinite as drying adjuvants also enabled the spray-drying of surfactin without any loss in surfactant activity (Barcelos et al., 2014).

Although biosurfactant drying feasibility through a spray-drying approach has already been demonstrated, to the best of our knowledge, the addition of drying adjuvants has always been required to recover the bioactive compounds. This addition of drying adjuvant is deleterious, as it decreases the purity of the product, enhances the cost, and increases the storage volumes. In this study, we investigate for the first time and in a systemic way the spray-drying of the three families of LPPs. The methodology that enables the drying of *B. subtilis*-based biosurfactants without the addition of adjuvants is also presented. The molecules from the three main families of LPPs produced by *B. subtilis* (i.e., surfactin, mycosubtilin, and plipastatin) have been considered for spray-drying at three different steps of their purification process. The preservation of the surfactant and antimicrobial activities after drying was assessed for the three LPPs.

## Materials and Methods

### Lipopeptides Production

Surfactin, mycosubtilin, and plipastatin (LPPs) were produced in 5-L shake flasks filled at 20% with Landy media, under orbital agitation at 160 rpm, as previously reported (Coutte et al., 2010a). Surfactin and plipastatin were respectively produced by *B. subtilis* BBG131 strain (Coutte et al., 2010a) and *B. subtilis* Bs2504 strain (Ongena et al., 2007). BBG131 and Bs2504 were grown for 72 h at 37°C in a modified Landy MOPS culture medium (composition: 20 g·L^−1^ of glucose, 5 g·L^−1^ of glutamic acid, 1 g·L^−1^ of yeast extract, 1 g·L^−1^ of K_2_HPO_4_, 0.5 g·L^−1^ of MgSO_4_·7H_2_O, 0.5 g·L^−1^ of KCl, 16 mg·L^−1^ of l-tryptophan, 1.6 mg·L^−1^ of CuSO_4_·5H_2_O, 1.2 mg·L^−1^ of MnSO_4_·H_2_O, 0.4 mg·L^−1^ of Fe_2_(SO_4_)_3_·7H_2_O, and 21 g·L^−1^ of 3-morpholinopropane-1-sulfonic acid MOPS, buffered at pH 7.0 using KOH 3 M). Mycosubtilin was produced by *B. subtilis* BBG125 strain (Béchet et al., 2013). BBG125 was grown for 72 h at 30°C in another modified Landy MOPS culture medium (Guez et al., 2021) (composition: 20 g·L^−1^ of glucose, 2 g·L^−1^ of glutamic acid, 1 g·L^−1^ of yeast extract, 1 g·L^−1^ of K_2_HPO_4_, 0.5 g·L^−1^ of MgSO_4_·7H_2_O, 0.5 g·L^−1^ of KCl, 2.3 g·L^−1^ of (NH_4_)_2_SO_4_, 32 mg·L^−1^ of CuSO_4_·5H_2_O, 100 mg·L^−1^ of MnSO_4_·H_2_O, 30 mg·L^−1^ of Fe_2_(SO_4_)_3_·7H_2_O, and 21 g·L^−1^ of 3-morpholinopropane-1-sulfonic acid MOPS, buffered at pH 6.5 using KOH 3 M). In these culture conditions, BBG131, Bs2504, and BBG125 were able to produce respectively 1.2 ± 0.2 g·L^−1^ of surfactin, 0.4 ± 0.1 g·L^−1^ of plipastatin, and 0.05 ± 0.02 g·L^−1^ of mycosubtilin.

### Lipopeptides Purification Process

The different steps undertaken for the production and the purification of LPP from a *B. subtilis* fermentation are shown in Figure 1. At the end of the culture, the broth medium was centrifuged in an Avanti J-E centrifugation unit (Beckman Coulter, Krefeld, Germany) at 8,000 *g* for 40 min at 10°C, and the cells were discarded. The supernatant was then 10-times concentrated through a 10-kDa Hydrosart ultrafiltration membrane (Sartorius, Goettingen, Germany). The resulting retentate was then supplemented with demineralized water (4 volumes of water for 1 volume of retentate) in order to perform four successive diafiltration processes through the ultrafiltration membrane described above. The de-salted resulting retentate was subsequently supplemented with ethanol 100% (3 volumes of ethanol for 1 volume of diafiltered retentate) to reach a final concentration in ethanol at 75%. On the one hand, the addition of ethanol precipitates most of the proteins included in the retentate, and on the other hand, it leads to the disruption of the LPP micelles into free LPP molecules (with a molecular weight <10 kDa). The ethanolic fraction obtained was subsequently filtrated through the same membrane described above in order to separate the precipitate fraction (retentate) from the pre-purified LPP fraction (permeate). Finally, the ethanol contained in the pre-purified LPP fraction was evaporated using a rotary evaporator (Rotavapor^®^ R-300, Büchi Labortechnik AG, Flawil, Switzerland).

### Evaluation of the Lipopeptides Thermal Resistance

Before proceeding to LPP spray-drying, a preliminary experiment has been carried out to determine the resistance of surfactin, mycosubtilin, and plipastatin to high-temperature treatment. LPPs (surfactin, mycosubtilin, and plipastatin) formulated in powder form were supplied by Lipofabrik (Lipofabrik, Villeneuve d'Ascq, France). LPP powders were solubilized with demineralized water at a concentration of 1 g·L^−1^ for surfactin and plipastatin and 0.5 g·L^−1^ for mycosubtilin. LPPs were held in an aqueous solution, in a closed container avoiding evaporation, in a sand bath (VWR, Fontenay-sous-Bois, France) heated at 20°C, 75°C, 92°C, 109°C, 126°C, 134°C, or 143°C for 1 min. Before and after the thermal treatment, the number of LPPs was quantified by ultra-high-performance liquid chromatography (UPLC) according to the method described in *Lipopeptide Quantification and Purity Determination*. LPPs were considered to be stable at the tested temperature when the same amount of LPPs was quantified before and after the thermal treatment.

### Spray-Dried Materials

The characteristics of all the spray-dried LPP samples (LPP concentration and volume of the solution) of this study are presented in Supplementary Table S1. For each modality, the experiment (production, purification, and spray-drying) was performed in triplicate. Two types of LPP samples were spray-dried: either already formulated LPPs in powder forms (Supplementary Table S1A) or LPPs in solution sampled at different steps of the LPP production and purification using membrane ultrafiltration workflow (Figure 1; Supplementary Table S1B). LPPs (surfactin, mycosubtilin, and plipastatin) formulated in powder form, supplied by Lipofabrik (Lipofabrik, Villeneuve d'Ascq, France), were resuspended with demineralized water before the spray-drying step. Surfactin, plipastatin, and mycosubtilin samples of various purities were sampled at different steps of the LPP purification process (A, untreated supernatant; B, concentrated and diafiltered LPP solution; C, enriched LPP solution after ethanol evaporation) before the spray-drying process. The addition of maltodextrin as an aid for spray-drying was also evaluated with mycosubtilin supernatant. Thus, supernatant from fermentation broth and containing 1 g·L^−1^ of mycosubtilin was also spray-dried after the addition of 2.5% and 5% w/v maltodextrin (Glucidex IT 12) supplied by Roquette (Lestrem, France).

### Spray-Drying Conditions

The spray-drying of the solutions was carried out in the Büchi Mini Spray Dryer B-290 (Büchi Labortechnik AG, Switzerland) configured with an inlet temperature of 140°C and exhaust temperature of 70°C. The pressure of the spraying air was at 40-mm water column, and the pumping flow was maintained at 8.5 ml·min^−1^. During the spray-drying procedure, inlet and outlet air characteristics (temperature, percentage of humidity, dew temperature, and wet bulb temperature) were analyzed using the Testo 625 psychrometer (Testo, Titisee-Neustadt, Germany). These values were used to establish a water balance as well as an energy balance.

### Analysis of the Spray-Drying Performances

In order to calculate the yield of the spray-drying process, the dry matter of the obtained powder was determined with a desiccator (Presica XM60, Presica Instruments AG, Dietikon, Switzerland) set on 102°C. The yield (*Y*) and the specific LPP yield (*Y*_*LPP*_), expressed in percent, were respectively calculated according to Eqs 1, 2:

$$Y=\;\frac{DM_{powder}\;*\;m_{powder}}{DM_{solution}\;*\;m_{solution}}$$ (1)

$$Y_{LPP}=\;\frac{P_{lipopeptide}\;*\;m_{powder}}{C_{lipopetide}\;*\;V_{solution}}$$ (2)

with the following parameters:

$DM_{powder}\;$ : the dry matter of the obtained spray-dried powder (%);

$m_{powder}\;$ : the mass of the obtained spray-dried powder (g);

$DM_{solution}\;$ : dry matter of the aqueous solution before spray-drying (%);

$m_{solution}\;$ : mass of the aqueous solution before spray-drying (g);

$P_{lipopeptide}\;$ : the LPP purity in the spray-dried obtained product (%);

$C_{lipopeptide}\;$ : the LPP concentration in the spray-dried solution (g·ml^−1^);

$V_{solution}\;$ : volume of the aqueous solution before spray-drying (ml).

### Analysis of the Spray-Dried Products (Activity Assays)

In order to ensure that the spray-drying process is not altering LPP properties, several activity assays were carried out on the obtained spray-dried products. The surfactant properties of the three kinds of spray-dried LPPs (surfactin, mycosubtilin, and plipastatin) were evaluated by measuring the surface tension according to the Du Nouÿ method using a tensiometer TD1 (Lauda, Lauda-Königshofen, Germany) as described previously (Leclère et al., 2006). This was carried out in distilled water at pH 8. The values were compared with the surface tension measured on LPP solutions before spray-drying using the same methodology. The surfactant properties of LPPs are considered to be conserved if the values measured after spray-drying fit with those measured before spray-drying.

Anti-*Legionella* activity of surfactin was performed in a microtiter plate to evaluate the minimal inhibitory concentration (MIC) of surfactin against *Legionella pneumophila* strains. The strains *L. pneumophila* 2-15-1 and 2-15-2 used in this study were isolated and identified from sanitary water by the Institut Scientifique de Service Public of Liège and kindly made available to us for this study. The MIC has been determined following the standard protocol (Loiseau et al., 2015). Briefly, 100 µl of sterile liquid Buffered Yeast Extract (BYE) broth have been dispensed in each well of a 96-well plate. Then, 9 two-fold dilutions of surfactin (before spray-drying and after spray-drying) in BYE broth were made starting at a concentration of 50 mg·L^−1^. Wells were inoculated by dispensing 5 µl of a 10^5^ CFU·ml^−1^ of bacterial culture in the growth phase. Wells containing only 100 µl of BYE broth and wells inoculated BYE broth were used respectively as negative and positive controls. Plates were incubated at 37°C for 48 h. The MIC has been determined at the lowest concentration where no visible growth is observed in the wells. The mean value of MIC was presented.

The antifungal activities of plipastatin samples were determined against *V. inaequalis* S575 by measuring the EC_50_ as recently described (Desmyttere et al., 2019). The mean value of EC_50_ was presented.

The antifungal activity of mycosubtilin was evaluated using the microwell dilution method in a 96-well microplate. *Saccharomyces cerevisiae* DSM1333 strain was grown overnight at 37°C on Potato Dextrose Agar (PDA), dispersed in water, adjusted to a final OD_600nm_ of 0.04, and used as an inoculum. Mycosubtilin samples were dissolved in dimethyl sulfoxide (DMSO) and then diluted in Roswell Park Memorial Institute (RPMI) 2× media to reach a final concentration of 0.4 g·L^−1^. Ninety-six-well microplates were prepared by dispensing 100 µl of RPM 2× media in all wells except in the negative control. Initially prepared mycosubtilin samples measuring 200 µl were added to the first wells. Two-fold serial dilutions of 100 µl were made from columns 1 to 10 in a concentration range from 0.8 to 400 mg·L^−1^. The inoculum measuring 100 µl was added to each well except in the negative control. Distilled sterile water measuring 100 µl was added to the negative control. The final volume of each well was 200 µl. The plate was incubated at 37°C for 12 h. After incubation, the lowest concentration of each extract showing no yeast growth or turbidity was taken as its MIC value. The mean value of MIC was presented.

For these three antimicrobial assays, mean values of MIC or EC_50_ and CIs (*α* = 0.05) were calculated from three technical and three biological replicates using R software nlstools package (R version 3.5.3, R Core Team, 2019).

### Lipopeptides Quantification and Purity Determination

The determination of the LPP concentration and purity in the samples before and after the spray-drying process was performed by UPLC. The LPPs in powder form, either those before spray-drying (surfactin, plipastatin, and mycosubtilin provided by Lipofabrik, Villeneuve d’Ascq, France) or those obtained after each spray-drying experiment, were solubilized in 100% methanol at a concentration of 1 g·L^−1^. Prior to being injected, all samples were centrifuged (10,000 *g*, 10 min). The high-concentrated samples such as those sampled at the late phase of the LPP purification process (diafiltered fraction and enriched LPP fraction) were diluted with demineralized water in order to be properly quantified by the apparatus. All the measurements have been done with an injection volume of 20 µl into an Interchim C18 column (UP5TP18-250/030 C18, Interchim, Montluçon, France) equipped on an ACQUITY UPLC system (Waters, Milford, MA, USA) coupled to a UV detector (detection at 215 nm). The solvents used, water of high-performance liquid chromatography (HPLC) grade with 0.1% trifluoroacetic acid (Solvent A) and acetonitrile of HPLC grade with 0.1% trifluoroacetic acid (Solvent B), were provided by Sigma Aldrich (St. Louis, MO, USA). The solvent flow was 0.6 ml·min^−1^ throughout the chromatography column.

To quantify surfactin, plipastatin, and mycosubtilin, the same analysis method was applied with the following gradient: from 0 to 5 min, 95% A/5% B; from 5 to 40 min, a linear gradient from 95% A/5% B to 0% A/100% B; from 40 to 45 min, 0% A/100% B; and from 45 to 56 min, 95% A/5% B. The concentration of the sample was determined by comparison with 98% surfactin, 95% iturin A, and 90% fengycin standards, all provided by Sigma Aldrich (St. Louis, USA). The elution time of surfactins, mycosubtilins (and iturin A), and plipastatins, under the above-described analysis conditions, were respectively 36–41, 24–26, and 27–33 min. The retention time and second derivative of the absorption spectrum between 200 and 400 nm were used to identify the eluted molecules (Empower Software, Waters).

The purity of the analyzed powders was determined by analogy with the corresponding LPP standard of known purity. Both the analyzed samples and the corresponding standard were solubilized at a concentration of 1 g·L^−1^ in 100% methanol. After analysis according to the method described above, the purity (*P*_*sample*_ in %) was determined according to the following equation (with *P*_*standard*_ as the purity of the standard in %; *A*_*sample*_ as the total specific area of the sample in µV·s^−1^; and *A*_*standard*_ as the total specific area of the standard in µV·s^−1^):

$$P_{sample}=\;\frac{P_{standard}\;*\;A_{sample}}{A_{standard}}$$

## Results and Discussion

### Impact of Spray-Drying on Lipopeptides Activities

Prior to considering spray-drying as an appropriate method to formulate LPPs in powder form, it is important to ensure that this operation is not negatively impacting LPP activities. First of all, evaluation of the thermal resistance of the LPPs of *B. subtilis* was carried out. Commercial powders of the three LPPs (i.e., surfactin, plipastatin, and mycosubtilin, provided by Lipofabrik (Lipofabrik, Villeneuve d'Ascq, France)) were exposed 1 min to different temperatures (up to 143°C) in order to evaluate their thermal resistance and then were quantified by UPLC. Regarding the applied temperature, any degradation of the three LPPs tested was observed after exposure (*data not shown*). This result enables us to confirm that the structures of these LPPs are preserved after a short-time exposure at high temperatures (up to 143°C). This observation is in line with other previous studies that have demonstrated that the surfactant properties of surfactin for instance are preserved after a short-time exposure at temperatures of up to 170°C (Barcelos et al., 2014). Then, these different commercial LPPs in powder form have also been resuspended in demineralized water and subsequently spray-dried. Activities of the obtained dried LPPs have then been evaluated and compared with the activities of the non-spray-dried initial samples. The surfactant activity has been monitored for each LPP, while anti-*Legionella* activity was measured for surfactin samples, and antifungal activities were evaluated for both mycosubtilin and plipastatin samples.

#### Lipopeptide Surfactant Activity

**FIGURE 2:**
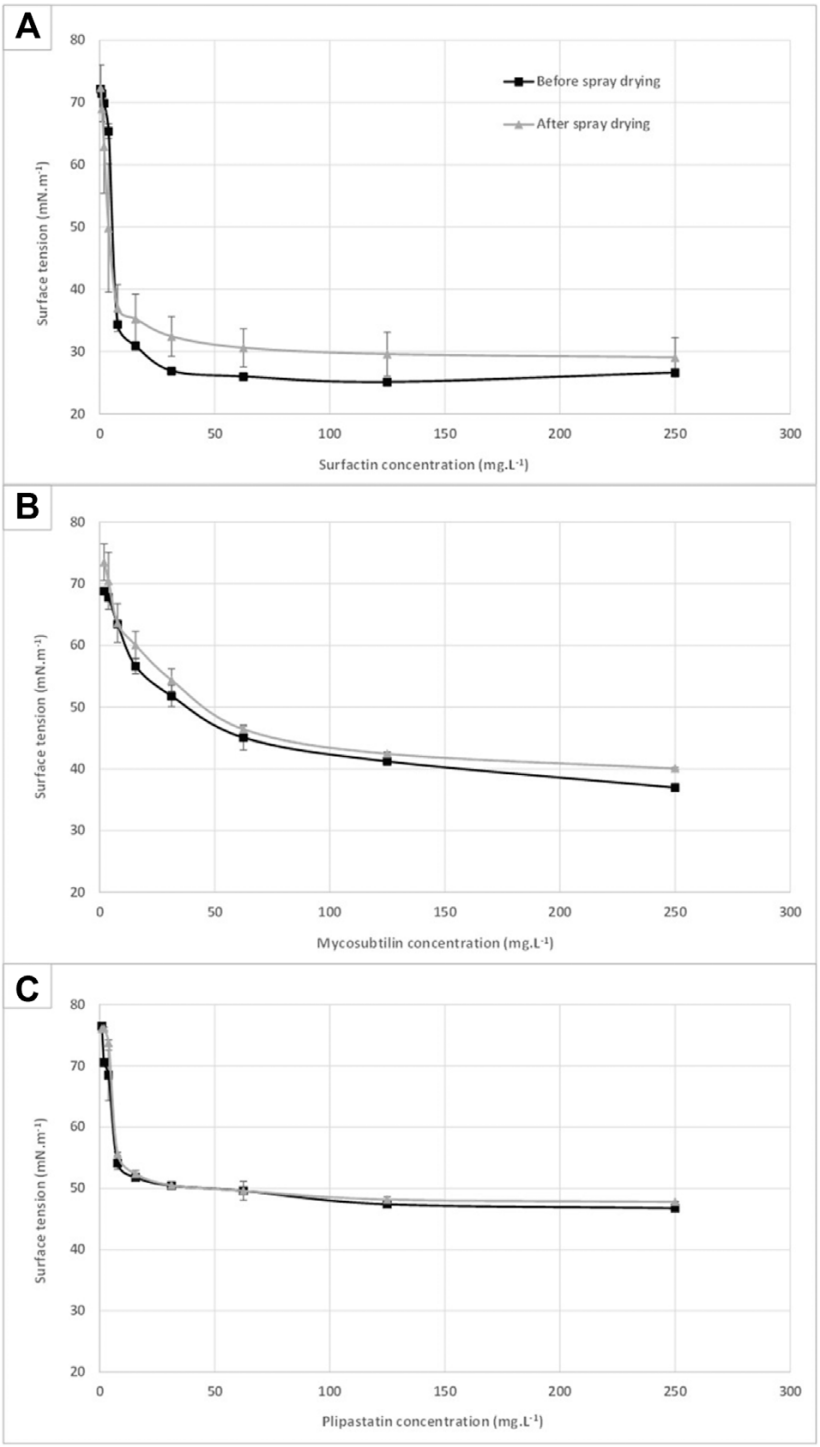
Surface tension profiles of surfactin **(** **A** **)**, mycosubtilin **(** **B** **)**, and fengycin **(** **C** **)** samples before and after the spray-drying process. Each value is an average based on three distinct samples measured in triplicates according to the Du Nouÿ method.

In order to assess the preservation of the biosurfactant activities of the three spray-dried LPPs, a surface tension measurement was performed according to the Du Nouÿ method on samples before and after the drying process. The results obtained are presented in Figure 2 for each LPP: surfactin (A), mycosubtilin (B), and plipastatin (C). No significant differences can be highlighted between the surface tension of mycosubtilin and plipastatin before and after spray-drying. Critical micellar concentrations (CMCs) of these molecules were calculated and are presented in Table 1 in comparison with the values found in the literature. The micellization process of these amphiphilic molecules is dependent on the chemical structure (chemical composition, length, and isomery of the fatty acid chain) and on environmental parameters of the study (buffer, pH, and temperature) (Deleu et al., 2013). In the scientific literature, CMC of the different LPPs was measured in different aqueous solutions (water or saline buffer) and was from 10 mg·L^−1^ for surfactin in pH 8.5 in 5 mM of Tris–HCl or in 0.1 M of NaHCO_3_ buffer (Thimon et al., 1992; Abdel-Mawgoud et al., 2008) to 25 mg·L^−1^ in water (Cooper et al., 1981), from 39 mg·L^−1^ in 0.1 M of NaHCO_3_ (Thimon et al., 1992) to 44 mg·L^−1^ for mycosubtilin in KCl 0.1 M (Maget-Dana et al., 1992) and from 2 mg·L^−1^ (Eeman et al., 2014) to 4 mg·L^−1^ for fengycin (plipastatin) in PBS buffer (Mantil et al., 2019). A slight impact can be observed on the surface tension profile of the surfactin with a surface tension approximately 10% higher in the surfactin samples, which has undergone a spray-drying step, from the concentration of 10 mg·L^−1^. Nonetheless, the biosurfactant properties of the spray-dried surfactin samples have been preserved. The lower CMC value of mycosubtilin obtained here could be explained by the nature of the isoforms present, probably different from those tested in the literature, but also the difference in the ionic strength of the solution assay.

**TABLE 1:** Critical micellar concentration of the lipopeptides produced by *Bacillus subtilis* before and after spray-drying process.

Sample type	CMC (mg·L^−1^) before spray-drying	CMC (mg·L^−1^) after spray-drying	CMC (mg·L^−1^) from literature	References
Surfactin	10.1	8.4 ± 0.7	10 – 25	Cooper et al. (1981); Thimon et al. (1992); Abdel-Mawgoud et al. (2008)
Mycosubtilin	27.2	26.8 ± 1.5	39 – 44	Maget-Dana et al. (1992); Thimon et al. (1992)
Plipastatin	9.2	10.2 ± 0.8	2 – 4	Eeman et al. (2014); Mantil et al. (2019)

Note. CMC, critical micellar concentration.

#### Lipopeptide Antimicrobial Activity

An antagonist antimicrobial assay was performed in order to determine whether the spray-drying procedure has an impact on the antibacterial activity of surfactin and on antifungal activities of mycosubtilin and plipastatin. The anti-*Legionella* activity of surfactin has been described for the first time several years ago by Loiseau et al. (2015). In this work, we investigated this specific antibacterial activity of surfactin against the opportunistic pathogen *L. pneumophila* before and after spray-drying. The results obtained are summarized in Table 2. The results obtained for surfactin against the two strains of *L. pneumophila* showed a different sensitivity of the LPP between them where the *L. pneumophila* 2-15-1 seems to be 1.6 times more sensitive than the strain *L. pneumophila* 2-15-2 (2.6–4.2 mg·L^−1^). The MIC values obtained against these two strains are in agreement with those published by Loiseau et al. (2015) where MIC values between 2 and 4 mg·L^−1^ were found against these pathogen species (Loiseau et al., 2015). The spray-drying process seems to slightly reduce the activity of surfactin, as the MIC obtained for the two *L. pneumophila* strains are approximately 1.2 times higher than the surfactin before spray-drying. However, this is not significant in terms of the different confidence intervals.

**TABLE 2:** Determination of the lipopeptide activities before and after the spray-drying process: anti-*Legionella* activity for surfactin samples **(A)**, antifungal activity against *Saccharomyces cerevisiae* for mycosubtilin samples **(B)**, and antifungal activity against *Venturia inaequalis* for plipastatin samples **(C)**.

Sample	A	MIC *L. pneumophila* 2-15-1 (mg·L^−1^)^a^	MIC *L. pneumophila* 2-15-2 (mg·L^−1^)^a^	B	MIC *S. cerevisiae* (mg·L^−1^)^a^	C	EC_50_ *V. inaequalis* (mg·L^−1^)^a^
Before spray-drying	Surf.	2.6 [1.6–3.6]	4.2 [2.1–6.2]	Myco.	4.1 [2.1–6.3]	Plip.	0.0124 [0.0062–0.0249]
After spray-drying		3.3 [1.9–4.7]	5.2 [3.4–7.0]		4.1 [2.1–6.3]		0.0077 [0.0066–0.0086]

Note. MIC, minimal inhibitory concentration.

a Mean value and CIs are presented from three biological and three technical replicates.

The antimicrobial potential of mycosubtilin and plipastatin was respectively assessed through the determination of the MIC for *S. cerevisiae* (Table 2) and the EC_50_ for *Venturia inaequalis* (Table 2). The obtained results enable us to conclude that the spray-drying process did not impact the antifungal potential of the mycosubtilin toward *S. cerevisiae*, as the same MIC of 4.1 mg·L^−1^ [2.1–6.3] was obtained before and after the spray-drying process. A slight impact of the drying process can be noticed on the antifungal potential of plipastatin, which seems to be increased after spray-drying, but this is not significant regarding CIs obtained for both samples (before and after spray-drying; see Table 2). Nonetheless, taken as a whole, the results demonstrate that the antimicrobial properties of both mycosubtilin and plipastatin have been maintained after the drying process.

### Performances of the Spray-Drying Process Regarding the Considered Purification Step

After demonstrating, on highly pure molecules, that spray-drying does not affect the activities of LPPs (surfactant and antimicrobial), we were interested in the performances of this drying process on different LPP preparations resulting from the different steps of the ultrafiltration purification process, namely, the cell-free supernatant, the diafiltered fraction, and finally the purified fraction. These different steps are presented in Figure 1. The dry matter of the spray-dried LPP solution and of the obtained powder, as well as the LPP purity in the dried product, is depicted in Table 3. From these first results, it can be concluded that spray-drying is a very efficient process to obtain LPPs in powder form with a high dry matter of 93.6% ± 2.2% whatever the considered family of LPPs is; nevertheless, the state of purification of the latter has an important impact.

**TABLE 3:** Dry matter values measured for each spray-dried lipopeptide sample on the liquid solution before drying (Sample) and on the dried powder obtained (Product).

Sample type		Sample DM (%)	Product DM (%)	Product purity (%)
Surfactin	A—Supernatant	3.14 ±0.13	94.45 ±1.27	3.4 ±0.5
	B—Diafiltered LPP fraction	0.81 ±0.07	95.55 ±0.72	58.8 ±2.2
	C—Enriched LPP fraction	0.62 ±0.02	96.48 ±0.23	82.4 ±1.0
Mycosubtilin	B—Diafiltered LPP fraction	1.51 ±0.45	93.63 ±0.89	8.7 ±1.8
	C—Enriched LPP fraction	0.61 ±0.10	90.94 ±1.60	63.1 ±10.0
Plipastatin	B—Diafiltered LPP fraction	0.67 ±0.02	93.22 ±0.54	31.0 ±5.4
	C—Enriched LPP fraction	0.52 ±0.05	90.72 ±1.62	97.7 ±4.0

Note. The lipopeptide purity in each spray-dried product is also indicated.

DM, dry matter; LPP, lipopeptide.

The results presented in Table 3 show that the purification process has a significant impact on the purity of the dried LPPs obtained. This result was expected from our previous work on this purification process (Coutte et al., 2010b; Jauregi et al., 2013). The purity obtained with diafiltered solution of surfactin (between 50% and 60%) using the same ultrafiltration procedure is in accordance with that previously published by our team (Coutte et al., 2013). Nevertheless, a small decrease in the performance of the last purification step (enriched LPP fraction) can be noticed for surfactin and mycosubtilin in comparison with the ones previously obtained (82.4% ± 1.0% and 78.3% ± 12.6%, respectively) (Coutte et al., 2013; Deravel et al., 2014), but this remains in very high values compared with other studies using the ultrafiltration process (Wei et al., 2010). Regarding the purification of plipastatin alone, there is little work in the scientific literature because it is often combined with surfactin (Coutte et al., 2010b; Coutte et al., 2017). Our results obtained using a cut-off of 10 kDa are satisfactory with respect to the literature, not only at the diafiltration step where the purity of more than 30% is obtained (Ben Ayed et al., 2015) but also after the last purification step. Indeed, a work using a combination of ultrafiltration, acid precipitation, and nanofiltration has shown that it was possible to obtain fengycin with a purity close to 95%, which is close to the purity of our enriched plipastatin fraction (97.7%) (Sen and Swaminathan, 2005). It should also be noted here that there is a significant difference in the purity of surfactin and plipastatin (or mycosubtilin) after the diafiltration step. This can be explained by the fact that the concentrations of the solutions are very different between these molecules (4.73 g·L^−1^ for surfactin, 1.22 g·L^−1^ for mycosubtilin, and 1.46 g·L^−1^ for plipastatin).

**FIGURE 3:**
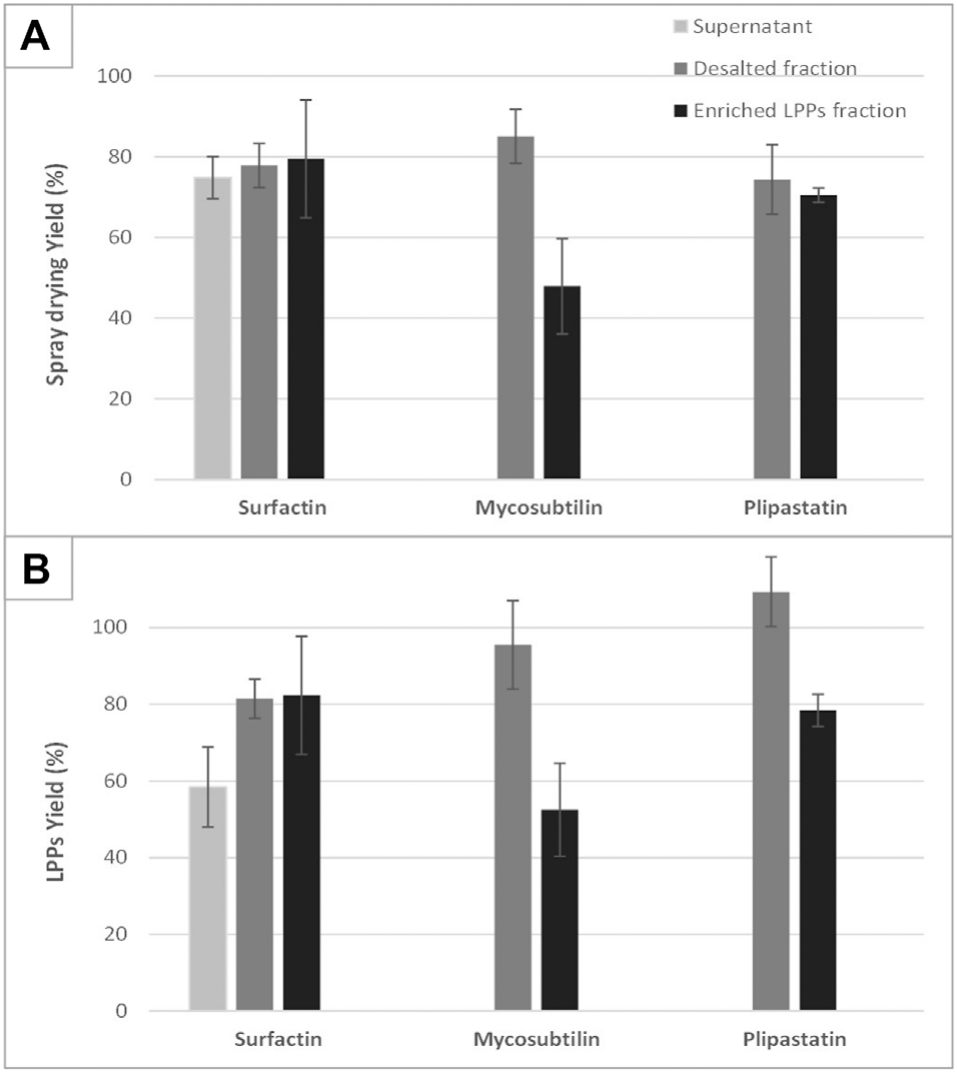
Overview of the performances of the spray-drying process in terms of process yield **(** **A** **)** and lipopeptide specific yield **(** **B** **)**. The drying performances of the three lipopeptides sampled at different steps of the purification process (supernatant, diafiltered fraction, and enriched lipopeptides fraction) are compared with each other and with reference samples corresponding to spray-dried and resolubilized commercial lipopeptides. Any analyzable dry product was obtained after spray-drying of mycosubtilin or fengycin supernatant. Each drying experiment was performed on three distinct samples coming from different production batches; mean values and standard deviation are presented.

The results presented in Figures 3A,B reveal, for the first time, that the yield of the spray-dried process is also related to the purity of the treated product. This is verified with spray-drying cell-free supernatant only, where the yield in LPP is quite low in the case of supernatant containing surfactin. For the latter two, a thick yellow paste is formed and sticks in an uncontrolled way all the parts of the spray-drier (data not shown). This phenomenon seems to be close to the one described by Barcelos et al. (2014), using crude LPP extract produced by *B. subtilis* LBBMA RI4914. A fermentation broth, even without cells, remains a complex matrix composed of numerous molecules that are more or less soluble in an aqueous solution (cell proteins, organic acids, biopolymers, sugars, etc.). This set of molecules (and particularly proteins) and the interactions they may have with each other when the solvent is removed can explain the formation of this sticky paste. Interestingly, in our study, this phenomenon does not appear when the supernatant contains only surfactin. Surfactin and plipastatin were produced by two strains derived from *B. subtilis* 168, thus showing a very similar potential of primary and secondary metabolite production profile (which is not the case for the mycosubtilin producing strain). Nevertheless, the overproduction of surfactin certainly limits the production of other metabolites by this strain, which may interfere with the spraying process. For surfactin, the yield of LPP recovered after the diafiltration step (diafiltered sample) is similar to the one obtained after the ethanol evaporation step (±80%). For plipastatin a slight decrease in the yield of LPP can be observed between the diafiltration and last purification steps (100%–78%). Nevertheless, for mycosubtilin, the yield of LPPs of the sample “purified fraction,” obtained after the ethanol evaporation step, is two times lower than the one obtained before this last step of purification (95% compared with 52%). This result seems to correlate with a decrease in the global spray-drying yield after this step of purification (Figure 3A). This is a surprising result, which led us to investigate deeper the case of mycosubtilin.

### The Specific Case of Mycosubtilin

**FIGURE 4:**
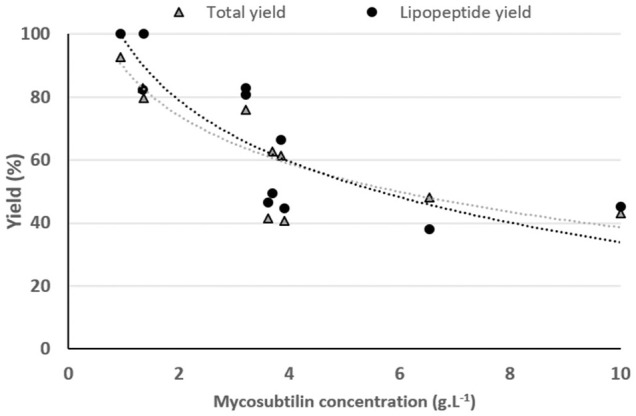
Impact of the mycosubtilin concentration on the total yield and the lipopeptide yield of the spray-drying process. Gray triangle, total yield; black circle, lipopeptide yield. Each drying experiment was performed on distinct samples coming from different production batches.

As shown in Figures 3A,B, a decrease in the yield of spray-drying of mycosubtilin after the last step of the purification process (ethanol evaporation) is observed. For a better understanding of this phenomenon, we studied the impact of the initial concentration of mycosubtilin in the solution to be spray-dried. Samples from 0.9 to 10 g·L^−1^ of mycosubtilin were spray-dried to mimic the concentration conditions obtained after the diafiltration or after the ethanol evaporation steps of purification. The results are presented in Figure 4. Interestingly, the total yield and the LPP yield decrease with the increase in the concentration of mycosubtilin, until a limit concentration of approximately 3.5 g·L^−1^. Indeed, results obtained for solutions below 3.5 g·L^−1^ are 90% ± 10% for LPP yield and 83% ± 5% for total yield. For solutions above 3.5 g·L^−1^, these results decrease to 50% ± 10% and 48% ± 10%. The ethanol evaporation process, which generates a high concentration, obviously has an important impact on the agglomeration phenomenon. The behavior of mycosubtilin in an aqueous solution is totally different from that of the other families of LPPs. It was shown that in the case of iturin A, micelles are formed at the CMC and larger vesicular structures at higher concentrations (Grau et al., 2001). In a more recent study, authors have investigated the behavior of the three families of LPPs using cryo-transmission electron microscopy, X-ray diffraction, and small-angle X-ray scattering. Their results confirm that mycosubtilin at high concentration has a distinct mode of self-assembly into extended nanotapes based on the stacking of LPP bilayers (Hamley et al., 2013). Moreover, Jauregi et al. (2013) have shown that mycosubtilin can interact with protein during the concentration/purification process by ultrafiltration (Jauregi et al., 2013). The amphiphilic, tensioactive, and uncharged nature of mycosubtilin and its ability to interact with proteins are important parameters to consider in a drying process. Indeed, it is likely to adsorb at the air–water droplet interface, where inadequate surface energies may expose the hydrophobic region and induce LPPs to aggregation, as it is well known for proteins (Lee, 2002; Ameri and Maa, 2007). To prevent this phenomenon, excipients/surfactants are commonly used for the drying process (especially for protein), but they also make the preparation stickier, which leads to a higher wall deposition and yield losses (Ameri and Maa, 2007). Knowing the propensity of mycosubtilin to agglomerate and its surfactant properties, it does not seem surprising to observe this wall deposition. Wall deposition is a key processing problem during spray-drying. This phenomenon is greatly impacted by the technical parameters of the apparatus (size and geometry of spray dryer, wall surface energy, etc.) and the operational conditions (inlet–outlet temperature, feed rates, and excipients) but not exclusively (Keshani et al., 2015). The impact of the concentration of the feed solution on the spray-drying efficiency has already been observed in the case of carbohydrates (Elversson and Millqvist-Fureby, 2005). Indeed, the concentration influences the particle size, with an increase in particle size until a plateau is reached. The authors also showed that the solubility of the carbohydrates had an impact on the particle size, the less soluble they were and the larger the particle size (Elversson and Millqvist-Fureby, 2005). Thus, these results show that the success of the spray-drying of this particular LPP is therefore also dependent on its concentration. It is advisable to limit its concentration below 3.5 g·L^−1^ to obtain a good yield.

Finally, to deepen your knowledge on the direct spray-drying of the supernatant containing mycosubtilin (at low concentration), some additional trials were run to determine if the addition of charge (i.e., maltodextrin) can enable to obtain a dry product from a mycosubtilin-based supernatant. Maltodextrin is commonly used in spray-drying as a drying aid; it is used during the process to reduce not only the thermoplasticity and hygroscopicity but also the stickiness and product deposition (Keshani et al., 2015; Lee et al., 2018). Two proportions of maltodextrin were evaluated: 2.5% and 5% (w/v). In both cases, an analyzable dry product was obtained with the addition of maltodextrin. With 5% w/v of maltodextrin, the mycosubtilin supernatant containing 6.64% of dry matter was dried into a powder at 96.57% dry matter, with 1.39% LPP purity, and with a process yield of 70.18% and an LPP yield of 67.1%. With 2.5% w/v of maltodextrin, the mycosubtilin supernatant containing 4.33% of dry matter was dried into a powder at 91.32% dry matter, with 2.15% LPP purity, and with a process yield of 88.05% and an LPP yield of 89.76%. In both cases, the antifungal properties of mycosubtilin were only slightly impacted (compared with the value obtained with purified mycosubtilin, i.e., 4.1 mg·L^−1^ [2.1–6.3]) with a MIC against *S. cerevisiae* of 5.2 mg·L^−1^ [3.1–7.3]. This confirms the results obtained by Barcelos et al. (2014), who showed that the addition of maltodextrin at 10% is effective for the spray-drying of supernatant of *B. subtilis*-containing LPPs. Nevertheless, we demonstrated that 4 times less maltodextrin is sufficient as filler to make the spray-drying of these supernatant-containing LPPs easier.

## Conclusion and Prospects

This study is the first one to systematically investigate the spray-drying of concentrated solutions of LPPs produced by *B. subtilis*. We have shown that this process produces a very high dry matter (up to 95%) and that surfactant and antimicrobial activities of the different LPPs are maintained. In addition, a more detailed study of the impact of the ultrafiltration purification process on the yield of the drying step demonstrated that this yield is directly impacted by the concentration of LPP, more particularly in the case of iturinic molecule such as mycosubtilin. Wall deposition phenomena were highlighted either when cell-free supernatants (for mycosubtilin and plipastatin) were used directly without additives or when the concentration of mycosubtilin was higher than 3.5 g·L^−1^. A specific study on the use of maltodextrin as an additive also showed its effectiveness for the direct drying of cell-free supernatants by limiting wall deposition phenomena. In the future, the impact of the operating conditions of the spray-drying process could be investigated (inlet temperature, feed concentration, and feed rate) in order to improve the yields of this process and constrain the phenomena of wall deposition. A granulometric study using microscopic means could also provide valuable information to characterize the formation of micellar aggregates, which can be involved in wall deposition phenomena.

## Data Availability

The original contributions presented in the study are included in the article/Supplementary Material. Further inquiries can be directed to the corresponding author.

## Abbreviations

CMC, critical micellar concentration; LPPs, lipopeptides; MIC, minimal inhibitory concentration; NRP, non-ribosomal peptide; NRPS, non-ribosomal peptide synthetase; PKS, polyketide synthase.
