# Supplementary material for: Impact of the Purification Process on the Spray-Drying Performances of the Three Families of Lipopeptide Biosurfactant Produced by Bacillus subtilis
Source: Front Bioeng Biotechnol. 2021 Dec 22;9:815337. doi: 10.3389/fbioe.2021.815337 (PMC8727909; doi:10.3389/fbioe.2021.815337)
Supplement: Supplementary file 1 [file Table1.pdf]

Table S1 : Overview of the different lipopeptide samples spray-dried in this study. **A.** Reference lipopeptide samples obtained after solubilization of powder-formulated lipopeptides provided by Lipofabrik (Lipofabrik, Villeneuve d'Ascq, France). **B.** Lipopeptide solutions sampled at different steps of the lipopeptide purification process. For each single sample (except mycosubtilin and fengycin supernatant), the spray-drying experiment was performed in triplicate, mean values and standard deviation are presented

A

Spray dried sample			Lipopeptide concentration in the spray dried solution ($g.L^{-1}$)	Volume of the spray dried solution (mL)
**Lipopeptide**	**Name**	**Powder purity (%)**		
Surfactin	Surf.90	90	10.09 ± 0.24	500
Mycosubtilin	Myco.67	67	3.23 ± 0.02	240
Plipastatin	Plip.98	98	4.33 ± 0.04	240

B

Spray dried sample		Lipopeptide concentration in the spray dried solution ($g.L^{-1}$)	Volume of the spray dried solution (mL)
**Lipopeptide**	**Corresponding process step**		
Surfactin	**A** - Supernatant	1.43 ± 0.02	1608.66 ± 60.05
	**B** – Diafiltered lipopeptide solution	4.73 ± 0.22	488.67 ± 29.02
	**C** - Enriched lipopeptide fraction (after ethanol evaporation)	5.10 ± 0.11	312.00 ± 4.58
Mycosubtilin	**A** - Supernatant*	0.05	2931
	**B** - Diafiltered lipopeptide solution	1.22 ± 0.24	498.55 ± 15.34
	**C** - Enriched lipopeptide fraction (after ethanol evaporation)	3.80 ± 0.16	195.28 ± 5.46
Plipastatin	**A** - Supernatant*	0.41	2089
	**B** – Diafiltered lipopeptide solution	1.46 ± 0.05	471.73 ± 5.15
	**C** - Enriched lipopeptide fraction (after ethanol evaporation)	5.05 ± 0.05	138.98 ± 17.92

* Any analyzable dry product were obtained after spray-drying of mycosubtilin or fengycin supernatant
